# The different responses of AOA and AOB communities to irrigation systems in the semi-arid region of Northeast China

**DOI:** 10.3389/fmicb.2024.1374618

**Published:** 2024-05-07

**Authors:** Ruowen Qiang, Meng Wang, Qian Li, Yingjie Li, Cuilan Li, Jinjing Zhang, Hang Liu

**Affiliations:** ^1^Key Laboratory of Soil Resource Sustainable Utilization for Jilin Province Commodity Grain Bases, College of Resource and Environmental Science, Jilin Agricultural University, Changchun, China; ^2^Institute of Agricultural Environment and Resources Research, Jilin Academy of Agricultural Sciences, Changchun, China

**Keywords:** mulched drip irrigation, AOA, AOB, community composition, symbiotic network

## Abstract

Ammonia oxidation is the rate-limiting step in nitrification and the key step in the nitrogen (N) cycle. Most soil nutrients and biological indicators are extremely sensitive to irrigation systems, from the perspective of improving soil fertility and soil ecological environment, the evaluation of different irrigation systems and suitability of selection, promote crop production and soil quality, study the influence of the soil microenvironment contribute to accurate evaluation of irrigation farmland soil health. Based on the *amoA* gene, the abundance and community diversity of ammonia-oxidizing archaea (AOA) and ammonia-oxidizing bacteria (AOB) and their responses to soil physicochemical factors and enzyme activities were studied in semi-arid areas of Northeast China. The study consisted of three irrigation systems: flood irrigation (FP), shallow buried drip irrigation (DI), and mulched drip irrigation (MF). The results showed that DI and MF significantly increased the contents of alkaline hydrolyzed nitrogen (AN), nitrate nitrogen (NO_3_^–^-N), soil moisture, and the activities of ammonia monooxygenase (AMO) and hydroxylamine oxidase (HAO). Compared with FP, DI significantly increased the abundance of soil AOA and AOB, while MF significantly increased the abundance of soil AOB. Irrigation systems significantly affected the community composition of ammonia-oxidizing microorganisms (AOM). Also, AN and soil moisture had the greatest influence on the community composition of AOA and AOB, respectively. The AOB community had better stability and stress resistance. Moreover, the symbiotic network of AOB in the three irrigation systems was more complex than that of AOA. Compared with FP, the AOA community under treatment DI had higher complexity and stability, maintaining the versatility and sustainability of the ecosystem, while the AOB community under treatment MF had higher transfer efficiency in terms of matter and energy. In conclusion, DI and MF were more conducive to the propagation of soil AOM in the semi-arid area of Northeast China, which can provide a scientific basis for rational irrigation and N regulation from the perspective of microbiology.

## 1 Introduction

Nitrogen (N) is an important nutrient for plant growth and plays an indispensable role in agro-ecosystems. Nitrification and denitrification processes are the main processes of the N cycle, both of which are driven by microorganisms. The composition and function of microbial communities in the N cycle can affect the bioavailability of N and thus regulate the growth characteristics of crops (Yu et al., 2019). The nitrification process plays a key role in the global N cycle, where ammonia nitrogen oxides into nitrite and nitrite oxides into nitrate. The nitrite oxidation is a two-step process that is respectively catalyzed by ammonia-oxidizing microorganisms (AOM) and nitrite oxidizing bacteria (NOB). The AOM includes ammonia-oxidizing archaea (AOA) and ammonia-oxidizing bacteria (AOB) and both AOB and AOA contain *amoA* genes which are often used as molecular markers to study the diversity and abundance of AOM in the natural environment (Tolar et al., 2017; Yao et al., 2022). The *amoA* gene catalyzes the conversion of NH_3_ to NO_2_^–^. It is an intracellular enzyme, including three subunits *amoA*, *amoB*, and *amoC*. AOB and AOA are widely distributed, and their relative abundance is closely related to the concentration of ammonia nitrogen. Petersen et al. (2012) found that the abundance of functional genes of AOM is the most important variable in predicting the potential rate of nitrification in soil. AOA and AOB coexist in most soils, and AOA is usually more abundant than AOB, hence AOA is considered to be the dominant microorganism in the soil ammonia oxidation process (Xiao et al., 2021). However, some studies have contradictorily indicated that the change in ammoxylation activity is only related to the abundance and composition of the AOB community, but not to the AOA community (Ouyang et al., 2016). With the continuous development of molecular biology, several studies have focused on understanding AOA and AOB communities (Xiao et al., 2021). At present, the niche differentiation and functional complementarity between AOA and AOB have been concluded (Lehtovirta-Morley, 2018). In addition, most studies have paid attention to the effects of different environmental conditions on AOA, AOB abundance, and community structure composition, as well as the relative contributions of AOA and AOB to nitrification (Ouyang et al., 2017; Szukics et al., 2019). Environmental factors such as pH, temperature, and N will all have a certain impact on the main role of AOA and AOB in the ammonia oxidation process, hence it is of great practical significance to further analyze the relative roles of AOA and AOB combined with environmental factors (Kou et al., 2023). Based on the previous research results, we found that different water management led to changes in the abundance and community structure of soil nitrifying microorganisms. However, under different environmental conditions, the response of these functional microorganisms to different water management varies (Guo et al., 2021; Wang and Huang, 2021). Therefore, studying the effects of different irrigation modes on soil AOM can help enrich our understanding of soil N cycling and key microorganisms, and is of great significance for targeted regulation of soil N transformation processes. However, the diversity of AOA and AOB communities under different irrigation modes and their response mechanisms to soil physicochemical factors and enzyme activities remain bleak.

Farmland in the western part of Northeast China is widely distributed and is one of the major grain production areas. However, the damaged ecological environment, semi-arid climate, and interaction of human factors in this region restrict the sustainability of food production in this region to a certain extent (Gu et al., 2018; Xin et al., 2020). Some studies have confirmed that crop yield in semi-arid areas is especially dependent on flood irrigation (Zhang et al., 2017). Heavy irrigation with low water efficiency can cause nitrate to enter deeper soil and groundwater, or sometimes it is permanently lost (Beaudoin et al., 2021). Therefore, since the 1990s, various agricultural measures have been adopted to reduce flood irrigation and N fertilizer consumption and maintain high crop yields (Zhang et al., 2019a). Drip irrigation is an effective irrigation and fertilization measure that helps to improve the water and N utilization efficiency of crops (Fan et al., 2020). China continuously promotes and optimizes water-saving irrigation technology in agricultural production. Drip irrigation, with its remarkable effect of saving water and increasing production and its eco-friendly features, has been rapidly promoted and applied as a model of efficient water-saving irrigation in semi-arid planting areas of northeast and western China. At present, common drip irrigation methods include under-film drip irrigation and shallow-buried drip irrigation (Li H. et al., 2021). With the characteristics of water retention, fertilizer saving, and high light efficiency, drip irrigation under film can achieve efficient water saving and effectively increase the accumulation of dry matter (Li et al., 2023). The shallow-buried drip irrigation method lacks film coverage, resulting in a decrease in soil water retention and a rapid decrease in soil temperature, culminating in an insignificant increase in production. However, the shallow-buried drip irrigation method can overcome the problems of premature aging, residual film pollution, and high cost of maintenance in the later growth stage of drip irrigation (Wang Z. et al., 2019). Irrigation is a key factor affecting agricultural ecosystems and it can shape the microbial community structure closely related to the N cycle in soil by changing the soil microenvironment. The growth of AOM is affected by the soil environment. Therefore, a reasonable irrigation mode can be determined by exploring the characterization of AOM in soil under different irrigation methods.

At present, the effects of different irrigation methods on soil ammonia-oxidizing microbial community structure are still unclear. Therefore, in this study, real-time qPCR and Illumina Miseqsequencing techniques were used to analyze the abundance, community composition, and diversity of soil AOM. This will help to identify the main driving factors of ammonia-oxidizing microbial community changes and clarify the mutual response of ammonia-oxidizing microbial community with soil nutrients and enzyme activities under different irrigation methods. The findings of this study will provide an important reference and theoretical basis for the application and popularization of drip irrigation technology in the semi-arid area of Northeast China.

## 2 Materials and methods

### 2.1 Experimental design

The test site was located in Minle Village, Ningjiang District, Songyuan City, Jilin Province (45° 26′ N,125° 88′ E). The rainfall period is normally from May to September. The test soil is the meadow soil and the field trial started in 2017, with three treatments set up. Treatment 1: traditional flooding irrigation (FP, all fertilizers were applied as base fertilizer at one time); Treatment 2: shallow buried drip irrigation (DI, 30% N fertilizer and 50% phosphate and potassium fertilizer were applied as the base fertilizer, and the remaining fertilizer was applied with water for 4 times in the later stage); Treatment 3: mulched film drip irrigation (MF, white transparent plastic film was covered on the corn and drip irrigation belt, 30% N fertilizer and 50% phosphorus and potassium fertilizer were applied as the base fertilizer, and the remaining fertilizer was applied with water for 4 times in the later stage). The N application rates for treatments DI and MF were pre-seeding (30%), jointing stage (30%), flowering stage (20%), silking stage (10%) and filling stage (10%), and the application rates for phosphate (P) and potassium (K) fertilizer were pre-seeding (50%), jointing stage (20%), flowering stage (15%), silking stage (10%) and filling stage (5%). The experimental maize variety was Xiangyu 998, and the planting density was 70 thousand hm^–2^. The same rates of N, P, and K were applied in all treatments, and the amount of irrigation was the same. The fertilizer dosage of N-P_2_O_5_-K_2_O was 210-90-90 kg⋅hm^–2^, the base fertilizer was urea (N 46%), diammonium phosphate (N-P_2_O_5_-K_2_O: 18-46-0), potassium chloride (K_2_O 60%). Topdressing was done using urea (N 46%), water-soluble fertilizer mono-ammonium phosphate (N-P_2_O_5_-K_2_O: 12-61-0), and water-soluble potassium chloride (K_2_O 60%). The size of each experimental plot was 10 m long, 3.6 m wide, and 36 m^2^ in area, and three repeated random plots were arranged. The experiment adopted a large-ridge double-row cultivation mode, with a narrow row spacing of 40 cm and a wide row spacing of 80 cm. The drip irrigation belt was laid in the middle of the narrow row during seeding, and the inner diameter of the drip irrigation belt was 16 mm with a drop head spacing of 30 cm. The source of water for the experiment was underground well water, and each district was connected to an independent fertilizer tank. During the fertilization process, the fertilizer tank was filled with the amount of fertilizer required by each district and thoroughly stirred for the fertilizer to be fully dissolved. Before fertilizing, the fertilizer tank valve was opened and the completely dissolved fertilizer was dropped. After fertilization, the water was allowed to continue dropping for 30 min.

### 2.2 Sample collection

In this study, soil samples were collected from the 0∼20 cm layer during the harvest period in 2021, and the soil samples from 5 randomly selected points were collected for each plot and mixed evenly to form a composite soil sample. After collection, the samples were returned to the laboratory in an ice box, and impurities such as roots and stones were removed, followed by passing the samples through a 2 mm sieve. Fresh soil samples were used for the determination of soil enzyme activity. Part of the soil samples were stored in a −80°C refrigerator at low temperature for the extraction of soil DNA, and the remaining soil samples were air-dried at normal temperature for the determination of soil physical and chemical properties.

### 2.3 Determination of physical and chemical properties of soil

Soil organic matter (SOM) was determined the by potassium dichromate oxidation and ferrous sulfate titration method; soil moisture was determined by the drying method; pH was measured by pH meter (water-to-soil ratio was 2.5:1); soil total nitrogen (TN) was determined by Kjeldahl method; soil alkali-hydrolyzed nitrogen (AN) was determined by diffusion method; soil ammonium nitrogen (NH_4_^+^-N) and soil nitrate nitrogen (NO_3_^–^-N) were determined by the continuous flow analyzer (Lu, 1999).

### 2.4 Determination of soil enzyme activities

Fresh soil samples (2 g) were extracted by ultrasonic wave for 10 min in 5 mL cell lysis solution (50 mmolL^–1^ Tris HCl (pH7.5), 10% (m/v) sucrose solution, 300 mmolL^–1^ sodium chloride and 90 mmolL^–2^ EDTA disodium salt). The extraction volume was adjusted to 25 mL with a buffer solution (pH 7.8) consisting of 50 mmolL^–1^ TrishCl and 5 mmolL^–1^ magnesium chloride. The extracts were centrifuged at 12000 r/min for 20 min, and enzyme activity was measured with supernatant. AMO activity was determined using the AMOELISA kit (SaintBio, Shanghai) according to the manufacturer’s instructions. The activity of HAO was measured by Abramyan et al. (1989).

### 2.5 Soil DNA extraction and fluorescence quantitative PCR

The total DNA was extracted from 0.5 g fresh Soil according to the FastDNA^®^ SPIN Kit for Soil (MP Biomedicals, USA) kit. The DNA concentration was determined by NanoDrop 2000 UV-VIS spectrophotometer (Thermo Scientific, USA), and the DNA quality was determined by 1% agarose gel electrophoresis. The primer sequences for the soil nitrification microbial *amoA* gene were A26F (5′-GACTACATMTTCTAYACWGAYTGGGC-3′) and A416R (5′-GGKGTCATRTATGG WGGYAAYGTTGG-3′) (Francis et al., 2005). The AOA *amoA* and AOB *amoA* genes were amplified with primer sequences *amoA*-1F (5′-GGGGTTTCTACTGGTGGT-3′) and *amoA*-1R (5′-CCCCTCKGSAAAGCCTTCTTC-3′), respectively (Chang et al., 2017). The reaction system was as follows: 10 μM upstream primer and downstream primer 1 μL, 1 μL SYBR^®^PremixExTaq™II (TliRNaseHPlus), ROXplusL, 25 μL 2 × TaqMasterMix, and 22 μL water, making a total of 50 μL. The PCR amplification conditions of the *amoA* gene were: predenaturation at 94°C for 5 min, 94°C 30 s, 55°C 30 s, 72°C 30 s, 30 cycles, and then extended at 72°C for 10 min after the last cycle was completed. Each sample was repeated 3 times, and the standard curve was established by soil ammonia-oxidizing microorganisms with known copy numbers.

### 2.6 Illumina Miseq sequencing and data analysis

The *amoA* gene was amplified using the total DNA extracted from soil microorganisms as a template. An 8 bp barcode sequence was added to the upstream 5′ end and downstream 3′ end of the primer in AOA *amoA* and AOB *amoA* to distinguish different samples. PCR reaction system (total system 25 μL): 12.5 μL 2× TaqPlusMasterMix, 3 μL BSA (2 ng μL^–1^), 1 μL ForwardPrimer (5 μM), 1 μL ReversePrimer (5 μM), 2 μL DNA (total added DNA was 30 ng), finally, 5.5 μL ddH_2_O was added to 25 μL. Reaction parameters: predenaturation at 95°C for 5 min, denatured at 95°C for 45 s, annealed at 55°C for 50 s, extended at 72°C for 45 s, 28 cycles, extended at 72°C for 10 min. The PCR products were detected by 1% agarose gel electrophoresis and purified by EncourAGee TampureXP nucleic acid purification kit. The PCR products were used to construct the microbial diversity sequencing library and were sequenced at Beijing Ovison Gene Technology Co., LTD.

The disembarkation data is segmented by QIME (V1.8.0) software according to the barcode sequence, and Pear (V0.9.6) software was used to filter and concatenate the data. Scores that were lower than 20, containing ambiguous base and primer mismatches were removed. The minimum overlap during splicing is set to 10 bp and the mismatch rate was 0.1. The Denovo method of Vsearch (V2.7.1) software was used to remove short sequences and chimeric sequences. The Vsearch UPARSE algorithm (V2.7.1) software was used to process the classification unit OTU (Operational Taxonomic Units) for high-quality sequence clustering, and the similarity threshold was 97%. The Blast algorithm was used to compare GenBank non-redundant nucleotide databases (nt) and a threshold of 1e−5 was set to obtain a species taxonomic information corresponding to each OTU. All raw sequences generated in this study have been deposited in NCBI under the accession SRP479978.

### 2.7 Statistical analysis methods

Using SPSS 23.0, *F* test in one-way ANOVA, multiple comparison of means (*P* < 0.05) was conducted with a Fisher’s protected least significant difference (LSD) in statistical analysis in soil physical and chemical properties, soil enzyme activities, and microbial diversities of ammonia oxidation among the different treatments. Pearson correlation analysis among soil physicochemical properties, soil enzyme activities, and abundance was performed using SPSS (V23.0). Canoco (V5.0) software was used for redundancy analysis (RDA) to elucidate the relationship between soil enzyme activities and physicochemical properties. Heatmap was done using R (V3.4.2) ‘pheatmap’ and ‘ggplot2’ packages (R Core Team, 2016). Principal component analysis (PCoA) and Anosim were performed using the Bray-Curtis algorithm in R software (V3.4.2). The ternary graph was created using “ggplot2” in R (V3.4.2). The structural equation model (SEM) was constructed by Amos (V23), chi-square test (*P* > 0.05), degree of freedom chi-square ratio (CMIN) / DF < 3, fitness index (CFI > 0.9). The visualization of cooccurrence network analysis was done by using the “Phych” packet of R (V3.4.2) software to generate the species correlation matrix. In addition, Gephi (V0.9.2) was used to draw the microbial network map and calculate the topological parameters. The function of nodes in a co-occurrence network was determined by inter-module connectivity (Pi) and intra-module connectivity (Zi) representation. According to inter-module connectivity (Pi) and intra-module connectivity (Zi), nodes were classified into peripheral nodes (Zi < 2.5 and Pi < 0.62), connectors (Zi < 2.5 and Pi > 0.62), module hubs (Zi > 2.5 and Pi < 0.62) and network hubs (Zi > 2.5 and Pi > 0.62).

## 3 Results

### 3.1 Soil physicochemical properties and AMO and HAO activities

**FIGURE 1:**
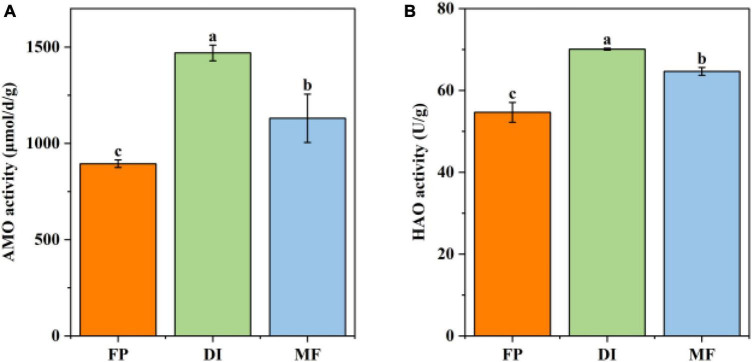
The soil AMO **(A)** and HAO activities **(B)** under different irrigation methods. Bars indicate SD, different letters indicate significant differences among samples (*P* < 0.05, ANOVA). AMO, ammonia monooxygenase; HAO, hydroxylamine oxidase; FP, traditional flooding irrigation; DI, shallow drip irrigation; MF, mulching drip irrigation.

Compared with FP, DI, and MF significantly increased the content of AN, NO_3_^–^-N, and soil moisture, and significantly decreased the content of NH_4_^+^-N. Compared with DI, MF significantly increased Moisture content, while pH and TN had no significant difference between different treatments (Table 1). The irrigation methods significantly changed soil enzyme activity. Compared with FP, DI and MF significantly increased the activities of AMO and HAO, where DI increased AMO and HAO activities by 1.64 and 1.28 times while MF increased by 1.26 and 1.18 times, respectively. Compared with DI, MF significantly reduced the activity of AMO and HAO by 1.3 and 1.03 times, respectively (Figure 1). The correlation between enzyme activities and soil physicochemical properties showed that the activity of HAO was positively correlated with AN (*r* = 0.862, *P* < 0.01), SOM (*r* = 0.723, *P* < 0.05) and NH_4_^+^-N (*r* = 0.783, *P* < 0.05). AMO was significantly positively correlated with AN (*r* = 0.810, *P* < 0.01), and SOM (*r* = 0.854, *P* < 0.01) (Supplementary Figure 1).

**TABLE 1:** Physicochemical properties of soil under different irrigation methods.

Treatment	pH	Moisture	SOM/	TN/	AN/	NH_4_^+^-N	NO_3_^–^-N
		(%)	(g/kg)	(g/kg)	(g/kg)	(mg/kg)	(mg/kg)
FP	6.75 ± 0.11a	10.84 ± 0.11c	17.06 ± 0.06b	1.05 ± 0.02a	95.54 ± 0.51b	0.66 ± 0.03a	7.93 ± 1.42b
DI	6.71 ± 0.35a	11.59 ± 0.45b	19.54 ± 0.71a	1.08 ± 0.02a	115.29 ± 1.63a	0.52 ± 0.07b	10.36 ± 1.11a
MF	7.04 ± 0.08a	13.81 ± 0.22a	17.81 ± 1.59ab	1.04 ± 0.03a	106.38 ± 8.06a	0.54 ± 0.01b	10.44 ± 1.03a

Different letters in the same column indicates a significant difference (*P* < 0.05, ANOVA). SOM, soil organic matter; TN, total nitrogen; AN, alkaline hydrolyzed nitrogen; NH_4_^+^-N, ammonium nitrogen; NO_3_^–^-N, nitrate nitrogen. FP, traditional flooding irrigation; DI, shallow drip irrigation; MF, mulching drip irrigation.

### 3.2 Abundance of soil ammonia-oxidizing microorganisms

**FIGURE 2:**
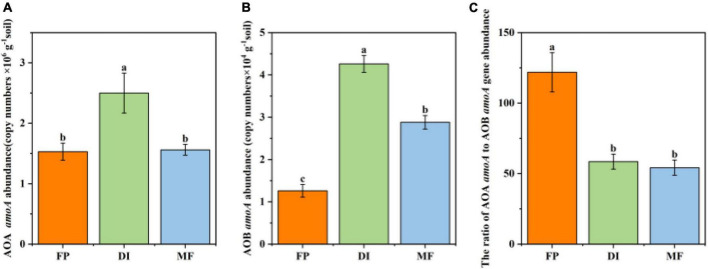
AOA **(A)**, AOB **(B)**, and AOA/AOB **(C)** *amoA* gene abundance under different irrigation methods. Bars indicate SD, different letters indicate significant differences among samples (*P* < 0.05, ANOVA). FP, traditional flooding irrigation; DI, shallow drip irrigation; MF, mulching drip irrigation.

Among the three irrigation methods, the abundance of AOA ranged from 1.53 to 2.60 × 10^6^ g^–1^ soil, and the abundance of AOB ranged from 1.26 to 4.26 × 10^4^g^–1^ soil, and the abundance of AOA was significantly higher than that of AOB (Figure 2). Compared with FP, DI significantly increased the abundance of AOA, 1.63 times than that of FP (*P* < 0.05) (Figure 2A). Compared with DI, MF significantly decreased the abundance of AOA, and DI was 1.60 times higher than that of MF (*P* < 0.05). Compared with FP, DI, and MF significantly increased the abundance of AOB by 3.4 times and 2.3 times, respectively (*P* < 0.05). Compared with DI, MF significantly decreased the abundance of AOB, and DI was about 1.48 times higher than that of MF (*P* < 0.05) (Figure 2B). The abundance ratio of AOA *amoA* and AOB *amoA* genes ranged from 58.64 to 121.16, which was not similar to the trend of AOA *amoA* and AOB *amoA* gene abundance. Compared with FP, DI and MF treatments significantly reduced the abundance ratio of AOA *amoA* and AOB *amoA* genes (Figure 2C). The correlation analysis of AOA and AOB abundance and soil physicochemical properties under three irrigation treatments showed that AOA abundance was significantly positively correlated with AN (*r* = 0.668, *P* < 0.05), SOM (*r* = 0.682, *P* < 0.05), and AOB abundance was significantly positively correlated with SOM (*r* = 0.710, *P* < 0.05), NH_4_^+^-N (*r* = 0.795, *P* < 0.05), AN (*r* = 0.911, *P* < 0.001) (Supplementary Figure 1). Correlation analysis of AOA and AOB abundance and enzyme activities under the three irrigation methods showed that AOA abundance was significantly positively correlated with AMO (*r* = 0.821, *P* < 0.01) and HAO (*r* = 0.737, *P* < 0.05), AOB abundance was significantly positively correlated with AMO (*r* = 0.931, *P* < 0.001) and HAO (*r* = 0.963, *P* < 0.001) (Supplementary Figure 1).

### 3.3 Community composition of soil ammonia-oxidizing microorganisms

**FIGURE 3:**
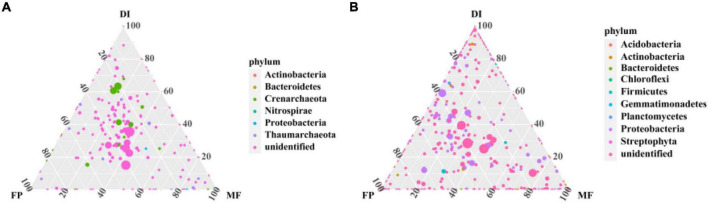
Phylum-level taxa of AOM communities uniquely associated with different irrigation methods. **(A)** Ternary phase diagram of AOA community at phylum level, **(B)** ternary phase diagram of AOB community at phylum level. The different colors in panels **(A,B)** represent different species, the dot size is proportional to relative abundance, and the closer the dot is to a vertex, the more abundant the species is in the group, with dotted grid intervals corresponding to a 20 percent increment. AOM, ammonia oxidizing microorganisms; FP, traditional flooding irrigation; DI, shallow drip irrigation; MF, mulching drip irrigation.

**FIGURE 4:**
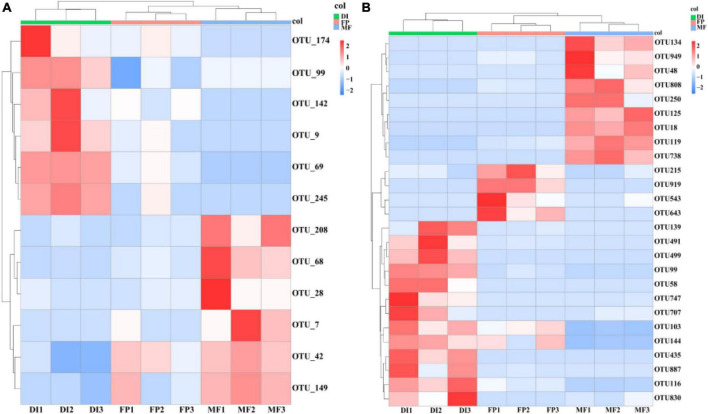
The relative abundance of dominant OTUs of soil AOM communities (**A** is AOA, **B** is AOB) under different irrigation methods. AOM, ammonia oxidizing microorganisms; FP, traditional flooding irrigation; DI, shallow drip irrigation; MF, mulching drip irrigation.

Sequencing from Illumina MiSeq, in order to analyze the microbial communities at the same sequencing depth, the lowest sequencing number of 73,184 sequences for AOA *amoA* gene and 30,160 sequences for AOB *amoA* gene were for randomly selected per sample. 799,214 high-quality sequences were obtained from all samples of the AOA *amoA* gene, each sample had 75,159–115,274 high-quality sequences, and the sequence length was optimized from 200 to 540 bp (Supplementary Table 1). Moreover, 385,327 optimized sequences were obtained from all samples of the AOB *amoA* gene, with 33,440 to 56,157 high-quality sequences per sample, and optimized sequence lengths of 200 to 540 bp (Supplementary Table 2). The OTU distribution among the samples revealed the OTU sharing of the microbiome in different irrigation treatments. The ternary phase diagram analysis of AOA and AOB based on three irrigation methods. In the ternary phase diagram of AOA, species were evenly distributed in the three treatments. The center of the triplet diagram is the influence of soil microbiome selection on the three irrigation treatments, and the core microbiome spans FP, DI, and MF treatments. FP recorded the highest relative abundance of Thaumarchaeota (80%), MF recorded the highest relative abundance of Proteobacteria (60%), and DI recorded the highest relative abundance of Crenarchaeota (80%) (Figure 3A). In the ternary phase diagram of AOB, the distribution of species in the three treatments was not very uniform. The relative abundance of Proteobacteria, Gemmatimonadetes, and Actinobacteria was higher in FP, DI, and MF treatments respectively (Figure 3B). Venn analysis revealed that FP, DI, and MF had 128 AOA OTUs and 988 AOB OTUs (Supplementary Figure 2). A total of 20 different OTUs were selected under AOA. Among them, OTU42 was the dominant bacterium with the relative abundance of 6.11–11.33%. Moreover, MF recorded a significant increase in the relative abundance of OTU42 (Figure 4A). BLAST analysis indicated that OTU42 (KM595440.1) might be an uncultured Nitrososphaerota (Supplementary Table 3). A total of 20 different OTUs were selected by AOB, among which OTU100 (MH589282.1) and OTU95 (KP212533.1) were the dominant bacteria, and their relative abundances were 10.64–14.75% and 7.81–11.87%, respectively. BLAST analysis showed that OTU100 (MH589282.1) could be uncultivated Nitrososphaerota and OTU95 (KP212533.1) may be uncultured *Nitrosospira* sp. (Supplementary Table 4). The heatmap showed that in AOA, compared with FP, DI significantly increased the relative abundance of OTU174, OTU99, OTU142, OTU9, OTU69 and OTU245 while MF significantly increased the relative abundance of OTU208, OTU68, OTU28, OTU7, OTU42, OTU42, and OTU149 (Figure 4A). In AOB, compared with FP, DI significantly increased the relative abundance of OTU139, OTU491, OTU499, OTU99, OTU58, OTU747, OTU707, OTU103, OTU144, OTU435, OTU887, OTU116 and OTU830, while MF significantly increased the relative abundance of OTU134, OTU949, OTU48, OTU808, OTU250, OTU125, OTU18, OTU119, and OTU738 (Figure 4B).

### 3.4 Diversity of soil ammonia-oxidizing microorganisms

**FIGURE 5:**
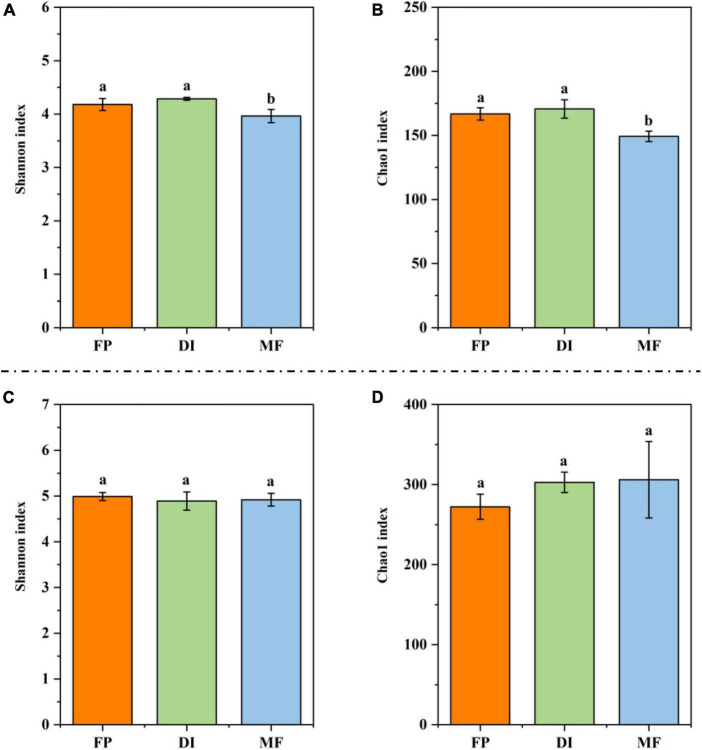
The alpha diversity for AOM communities under different irrigation methods. **(A)** Shannon index for AOA *amoA* gene; **(B)** Chao1 index for AOA *amoA* gene; **(C)** Shannon index for AOB *amoA* gene; **(D)** Chao1 index for AOB *amoA* gene. Bars indicate SD; different letters indicate significant differences among samples (*P* < 0.05, ANOVA). AOM, ammonia oxidizing microorganisms; FP, traditional flooding irrigation; DI, shallow drip irrigation; MF, mulching drip irrigation.

**FIGURE 6:**
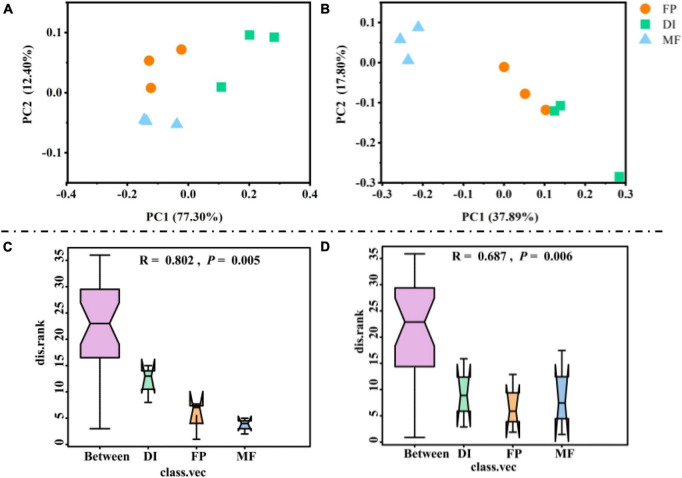
The beta diversity and Anosim analysis for AOM communities under different irrigation methods. PCoA of AOA **(A)**, AOB **(B)** communities based on OTU. Anosim analysis of AOA **(C)** and AOB **(D)** based on Bray-Curtis. AOM, ammonia oxidizing microorganisms; FP, traditional flooding irrigation; DI, shallow drip irrigation; MF, mulching drip irrigation.

Compared with FP and DI, MF significantly reduced the Shannon index and Chao1 index of AOA (Figures 5A, B). In AOB, the Shannon index and Chao1 index showed no significant difference in the different irrigation treatments (Figures 5C, D). The PCoA of soil AOM showed that for the PCA results of AOA, the contribution values of the first principal component and the second principal component were 77.30 and 12.40%, respectively, totaling 89.70%, reflecting the main reason for the difference among the three irrigation treatments. The relative distance between FP and MF treatment was relatively close, which was different from that of DI treatment (Figure 6A). The PCA results of AOB showed that the contribution values of the first principal component and the second principal component were 37.89 and 17.80%, respectively, totaling 55.69%, which was also the main reason for the difference among the three different irrigation treatments. The samples of FP and DI treatments were relatively clustered, and there was a difference between them and MF treatment (Figure 6B). In order to further determine whether the difference in soil AOA and AOB between groups and within groups was significant, Anosim analysis was performed on soil microbial colony structure, and the results showed that the difference between groups of the three irrigation treatments was significantly greater than the difference within groups (*P* < 0.05) (Figures 6C, D). In conclusion, irrigation methods significantly affected the community structure of AOA and AOB.

### 3.5 Environmental factors affecting soil ammonia-oxidizing microbial communities

**FIGURE 7:**
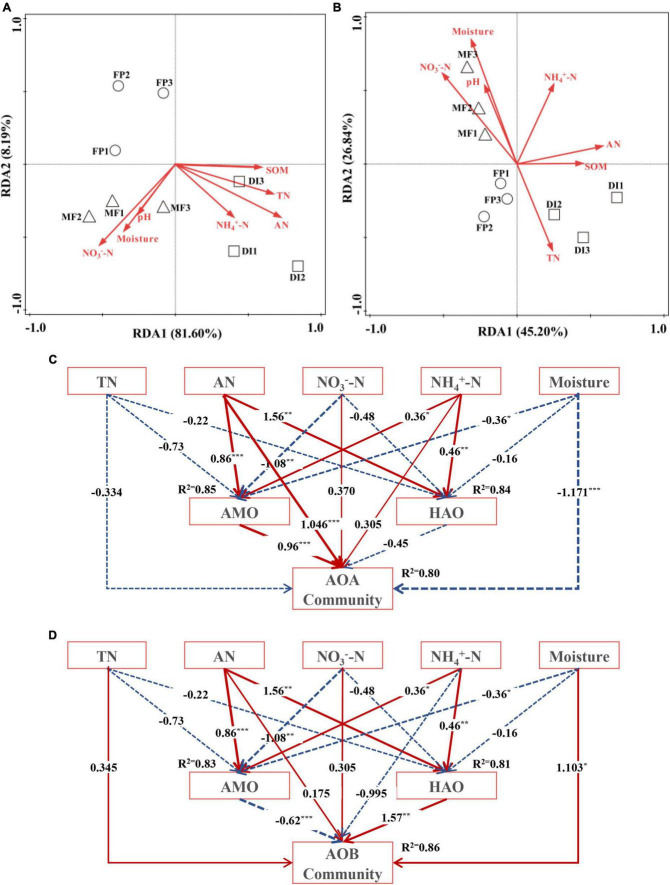
The RDA between AOM communities (**A** is AOA, **B** is AOB) and soil properties. The structural equation model (SEM) between the soil nutrient content, AMO, HAO, and AOA community **(C)**, AOB community **(D)**. The arrow width is proportional to the strength of the path co-efficients. Solid red arrows indicate a positive correlation, while blue dotted arrows indicate a negative relationship. *, ** and *** indicate *P* < 0.05, *P* < 0.01 and *P* < 0.001, respectively. SOM, soil organic matter; TN, total nitrogen; AN, alkaline hydrolyzed nitrogen; NH_4_^+^-N, ammonium nitrogen; NO_3_^–^_–_N, nitrate nitrogen; AMO, ammonia monooxygenase; HAO, hydroxylamine oxidase; AOM, ammonia oxidizing microorganisms; FP, traditional flooding irrigation; DI, shallow drip irrigation; MF, mulching drip irrigation.

RDA showed that in AOA, AN was the main driver of AOA community structural changes (Supplementary Table 5), and SOM, TN, AN, NH_4_^+^-N were positively correlated with DI. Moreover, pH, NO_3_^–^-N, and soil moisture were positively correlated with MF (Figure 7A). In AOB, soil moisture was the main driving factor of AOB community structural changes (Supplementary Table 6). The pH, NO_3_^–^-N and soil moisture were positively correlated with MF, while AN, SOM, TN, and NH_4_^+^-N were positively correlated with DI (Figure 7B). For AOA, SEM perfectly fitted the experimental data (χ^2^/df = 0.450, *P* = 0.77, CFI = 1.00), and the equation model explained 85% of the variation in AMO, 84% of the variation in HAO, and 80% of the variation in AOA community (Figure 7C). TN, AN, NO_3_^–^-N, NH_4_^+^-N and Moisture had direct effects on AMO, HAO and AOA communities, among which AN had the greatest effect on AMO (λ = 0.86, *P* < 0.001), HAO (λ = 1.56, *P* < 0.01) and AOA (λ = 1.046, *P* < 0.001) community. AMO and HAO had direct effects on AOA community, and AMO (λ = 0.96, *P* < 0.001) had the greatest effect on AOA community. For AOB, SEM fully fitted the experimental data (χ^2^/df = 1.119, *P* = 3.26, CFI = 0.998), and the equation model could explain 83% of the variation in the AMO, 81% of the variation in HAO, and 86% of the variation in AOB community (Figure 7D). TN, AN, NO_3_^–^-N, NH_4_^+^-N and Moisture had direct effects on AMO, HAO and AOB community, among which AN had the greatest effect on AMO (λ = 0.86, *P* < 0.001) and HAO (λ = 1.56, *P* < 0.01), Moisture (λ = 1.103, *P* < 0.05) had the greatest effect on AOB community. AMO and HAO had direct effects on AOB community, and HAO (λ = 1.57, *P* < 0.01) had the greatest effect on AOB community.

### 3.6 Symbiotic network analysis

**FIGURE 8:**
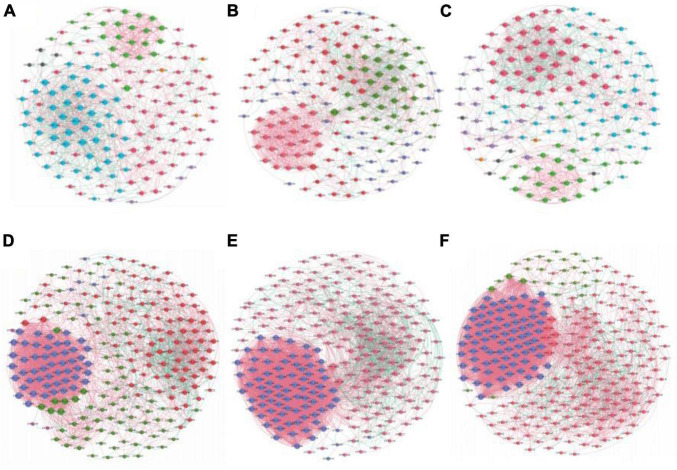
Co-occurrence network analysis of AOM communities under different irrigation methods. **(A)** AOA of FP, **(B)** AOA of DI, **(C)** AOA of MF, **(D)** AOB of FP, **(E)** AOB of DI, **(F)** AOB of MF. A connection represented a strong (Spearman’s correlation coefficient ρ > 0.7) and significant (*P* < 0.01) correlation. The thickness of each connection between two nodes, that is edge, is proportional to the value of Spearman’s correlation coefficients, the size of each node is proportional to the number of connections (degree).

The AOA and AOB symbiotic networks of the three irrigation treatments were used to test whether irrigation methods showed unique topological properties that affect microbial communities. Through the analysis of the average degree, clustering coefficient, average path length, complexity, density, and modularity characteristics of AOM networks, it was revealed that each network has some typical personalized modularity characteristics (Table 2). The AOA symbiotic network consisted of 153 (FP), 157 (DI), 144 (MF) nodes and 961 (FP), 1137 (DI), and 849 (MF) edges. These results showed that DI increased the number of nodes in the network compared to FP and MF. Also, DI increased the edge of the interaction of AOA species, the complexity of soil AOA networks, and enhanced the interactions between AOA species (Figures 8A–C). The AOB symbiotic network consisted of 241 (FP), 249 (DI), 276 (MF) nodes and 3034 (FP), 4442 (DI), 5514 (MF) edges. Compared with FP and DI, MF increases the number of nodes in the network and also increases the interaction edges of AOB species. MF increased the complexity of the soil AOB network and enhanced the interaction between AOB species (Figures 8D–F). Compared with AOA networks, AOB networks had the highest average degree, the shortest average path length, and the highest average clustering coefficient, indicating that AOB networks had the highest interspecific interaction degree and the highest connection tightness with closer OTUs connections.

**TABLE 2:** Network topological parameters of soil AOA and AOB communities under different irrigation methods.

Network properties	AOA co-occurrence network			AOB co-occurrence network		
	FP	DI	MF	FP	DI	MF
Nodes	153	157	144	241	249	276
Edges	961	1137	849	3034	4442	5514
Edge_density	0.0923	0.1049	0.0937	0.1049	0.1439	0.1453
Average degree	12.562	14.484	11.792	25.178	35.679	39.957
Average path length	3.068	3.047	3.159	2.764	2.599	2.694
Average_clustering_coefficient	0.524	0.533	0.506	0.582	0.619	0.632
Complexity	7.013	8.129	6.701	12.589	17.839	19.978
Modularity	0.525	0.547	0.533	0.319	0.347	0.468

FP, traditional flooding irrigation; DI, shallow drip irrigation; MF, mulching drip irrigation.

**FIGURE 9:**
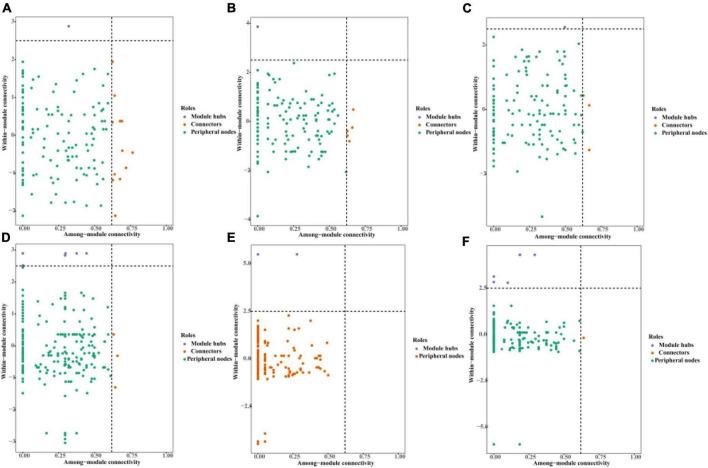
Topological role of AOM communities in different irrigation methods. The horizontal lines indicate 2.5 intra-module connectivity (Zi) and vertical lines indicate 0.62 inter-module connectivity (Pi). **(A)** AOA community of traditional irrigation; **(B)** AOA community of shallow drip irrigation; **(C)** AOA community of covered drip irrigation; **(D)** AOB community of traditional irrigation; **(E)** AOA community of shallow drip irrigation; **(F)** AOB community of covered drip irrigation.

Most of the nodes in the AOA and AOB symbiotic networks under the three irrigation modes were peripheral nodes, and none of the nodes in the AOA and AOB networks fell within the network hub. In the AOA symbiotic network: FP had 1 node in the module hub and 12 nodes in the connector; DI had 1 node in the module hub and 5 nodes in the connector; MF had 1 node in the module hub and 3 nodes in the connector (Figures 9A–C). In the AOB symbiotic network: FP had 6 nodes in the module hub and 3 nodes in the connector; DI had 1 node in the module hub; MF had 5 nodes in the module hub and 1 node in the connector (Figures 9D–F).

## 4 Discussion

### 4.1 Irrigation methods on soil physical and chemical properties

Compared with FP, DI and MF significantly increased soil water content. Qu et al. (2019) also indicated that mulching film significantly increased soil water content in arid and semi-arid areas. This may be due to the slow and uniform immersion of soil water into the soil during drip irrigation, which keeps the soil water-holding capacity relatively suitable and stable while not destroying the soil structure, thereby reducing the evaporation of soil water (Liu and Xu, 2023). To a certain extent, mulching film mitigates the impact of air convection, increases temperature, and preserves soil moisture, thus inhibiting the evaporation of surface water. This improves soil hydrothermal conditions and promotes the early growth of crops while saving water and fertilizer, thereby improving water use efficiency (Yu et al., 2018). There was no significant difference in SOM under different irrigation modes, which was consistent with the study by Núez et al. (2022). In this study, there was no significant difference in TN content among the three irrigation methods, which may be due to the dynamic balance of N in the soil (Lu et al., 2021). Compared with FP, DI, and MF significantly increased the content of AN in soil, which may be because the content of AN in the soil is not stable enough and is easily affected by soil hydrothermal and biological activities. Mulching increased soil temperature, intensified microbial activity, promoted nutrient release, and increased the content of AN (Wang et al., 2021a; Araujo et al., 2023). Compared with FP, DI and MF significantly increased NO_3_^–^-N content, while decreasing soil NH_4_^+^-N content, which may be due to faster evaporation of soil water after drip irrigation, better soil aeration, and soil surface N accumulation under film mulching treatment (Chen et al., 2020). Moreover, the film coating can slow down the downward migration of NO_3_^–^-N, leading to an increase in NO_3_^–^-N content under mulched drip irrigation (Wang et al., 2022). With the increase in soil aeration and soil temperature, the activity of soil nitrifying bacteria was enhanced, and the nitrification reaction was intensified, resulting in decreases in NH_4_^+^-N content (Li et al., 2022).

### 4.2 Influence of irrigation methods on the abundance of ammonia-oxidizing microorganisms

This study showed that the abundance of AOA *amoA* gene was significantly higher than that of AOB *amoA* gene, which was consistent with earlier studies that indicated that AOA *amoA* gene abundance dominates in some weakly acidic soils (Sun et al., 2019; Yao et al., 2022). Compared with FP, DI significantly increased the abundance of AOA *amoA* and AOB *amoA* genes, and MF significantly increased the abundance of AOB *amoA* gene. Drip irrigation promotes the nitrification of soil such that under drip irrigation conditions, water droplets slowly enter the soil and distribute around crop roots under the action of capillary force and gravity. In this region, the nitrification effect is higher than the denitrification effect (Sarula et al., 2023), thus increasing the abundance of soil AOA *amoA* and AOB *amoA* genes. In this study, the correlation between soil physicochemical properties and the abundance of AOA *amoA* and AOB *amoA* genes was studied by Pearson correlation analysis. The results showed that the soil AOA *amoA* abundance was positively correlated with AN and SOM, and the soil AOB *amoA* abundance was positively correlated with AN, SOM, and NH_4_^+^-N. Studies have shown that AOA is more adaptable to environmental conditions with low available ammonium N concentration, and plays a leading role in the nitrification process than AOB (Lin et al., 2021). On the contrary, AOB is more dominant in the nitrification process of neutral soils with high alkaline and N abundance (Wang et al., 2021b). The results indicated that the change of NH_4_^+^-N content had a more obvious effect on AOB abundance due to the different irrigation methods.

### 4.3 Effects of irrigation methods on the composition of ammonia-oxidizing microbial communities

At the phylum level, soil AOB community composition was significantly affected by irrigation methods, while soil AOA community changes were less affected. This may be due to the fact that the ecological niche of soil AOA is smaller than that of AOB, that is, it occupies a narrower range of resources and space in the soil, while soil AOB has a wider ecological niche and can survive under different conditions such as temperature, pH and N elements (Guo et al., 2023). Compared with FP, DI, and MF significantly changed the community structure and abundance of AOM in soil. The dominant microorganisms are relatively abundant in soil and play an important role in the regulation of ecological functions. The results of this study indicated that Thaumarchaeota, Crenarchaeota, and Proteobacteria are the dominant phyla in AOA. Thaumarchaeota is a typical ammonia-oxidizing archaea, and some studies have suggested that Thaumarchaeota plays an important role in the ammonia-oxidizing process (Yang et al., 2018; Wang N. et al., 2019). The significant increase in the relative abundance of Thaumarchaeota in FP may be due to the application of N fertilizer. Araujo et al. (2023) showed that Thaumarchaeota is the dominant archaea under N application systems. The research of Luo et al. (2023) also showed that Thaumarchaeota is significantly positively correlated with soil NH_4_^+^-N content and other related indicators, suggesting that Thaumarchaeota can accelerate catalytic ammonia oxidation to obtain energy for autotrophic growth (Wu et al., 2020). Studies have shown that most OTUs in AOA belong to Crenarchaeota (Wang and Huang, 2021), and an increase in the relative abundance of Crenarchaeota is conducive to promoting the rate of soil ammonia oxidation. An appropriate combination of fertilization and irrigation frequency can regulate soil AOA community composition, and the physicochemical properties also have different effects on Crenarchaeota. The highly relative abundance of Crenarchaeota in DI treatment may be due to the high SOM content and better soil fertility. Proteobacteria have a wide ecological range and strong suitability and can form a relatively stable ecological niche in different environments (Jiang et al., 2021). The highly relative abundance of Proteobacteria in MF may be due to the difference in soil water content. The findings of this study are consistent with Chen et al. (2020) who reported that Proteobacteria propagated in large numbers under high soil-water conditions.

In AOB, Firmicutes, Gemmatimonadetes and Actinobacteria are dominant bacteria. The *Bacillus* in Firmicutes produces spores that can resist drying, dehydration, and extreme environments. In the case of drought, spores can prolong the hydration of cells and provide nutrients to cells, so that cells can resist dehydration and other extreme environments (Li et al., 2020). The highly relative abundance of Firmicutes in FP treatment may be due to the fluctuations in moisture and the proliferation of these bacteria in low water content environments (Veach and Zeglin, 2020). Gemmatimonadetes was the dominant phylum of DI. Gemmatimonadetes can adapt to low-humidity environments, but cannot resist moisture fluctuations caused by dry and wet cycles. Moreover, studies have shown that Gemmatimonadetes is negatively correlated with soil organic matter (Zhang et al., 2019b). The highly relative abundance of Actinobacteria in MF may be due to the influence of SOM content. Javed et al. (2021) confirmed that the abundance of Actinobacteria bacteria can promote the formation of humus and decomposition of organic matter in soil, and also have a certain effect on the soil N cycle. As Actinobacteria tends to grow in soil with low SOM content, film mulching reduces the organic carbon content of rhizosphere soil, which is conducive to promoting the growth and propagation of Actinobacteria (Chen et al., 2017). According to Blast analysis, Nitrososphaerota is an important strain of AOA, and *Nitrososphaera* belongs to Nitrososphaerota. Previous studies have shown that the main AOA group in the black soil of Northeast China is *Nitrososphaera* (Ding et al., 2020), and it may be the main driving factor for nitrification of acidic soil in China (Li W. et al., 2021). *Nitrosospira* sp. was the dominant genus of AOB, which is consistent with previous studies on agricultural soil (Zhang et al., 2019c; Hu et al., 2021). *Nitrosospira* sp. produces nitrite by oxidizing soil nitrifying bacteria, which plays a crucial role in the nitrification process, making nitrogen easily absorbed by crops.

### 4.4 Environmental factors affecting the changes of ammonia-oxidizing microbial communities

In this study, the RDA of environmental factors and AOA community structure showed that AN was the main driving factor affecting change in the AOA community. The results of this study are consistent with the results of Sarula et al. (2023), who similarly reported that AOA community structure mainly depends on AN and pH. On the contrary, soil pH did not have much influence on the AOA community probably because there was little change in soil pH in this study. Sun et al. (2019) showed that pH plays an important role in shaping AOA community structure as similarly reported by other studies (Gubry-Rangin et al., 2011; Hu et al., 2013). Moreover, the RDA of environmental factors and AOB community structure showed that water content was the main driving factor affecting the change of the AOB community, which may be caused by the response of AOB to oxygen conditions. Similarly, Yang et al. (2018) showed that there was a significant correlation between the AOB community and soil water which indicates that soil water content significantly affects the community structure of nitrifying microorganisms in soil (Cong et al., 2023).

### 4.5 Influence of irrigation mode on the symbiotic network

In general, the higher the microbial community diversity index, the more complex the community structure and the better the stability (Jiang et al., 2020). The AOB symbiotic network was more complex than the AOA symbiotic network in the three irrigation methods, indicating that the three irrigation methods had better stability and stress resistance effects on the soil AOB community. This may be due to the significant increase in some key species in AMO in our study, thereby improving the network stability and connectivity of species in soil (Xiang et al., 2020). At the same time, the high stability of microbial communities is an important guarantee for the realization of ecological functions. Therefore, AOB plays an important role in enhancing the function of farmland soil under different irrigation methods. The study also identified key species of microbial communities by analyzing the topological structure of symbiotic networks, which change the composition of microbial communities (Zhou et al., 2021). In a microbial symbiotic network, nodes represent microbiota species in the biome, and the topological characteristics of different nodes can be used to determine the key species. Generally, node attributes are divided into four types; peripheral nodes (Zi < 2.5 and Pi < 0.62), connectors (Zi < 2.5 and Pi > 0.62), module hubs (Zi > 2.5 and Pi < 0.62), and network hubs (Zi > 2.5 and Pi > 0.62), where all nodes of the network hub are key species in the network. Nodes that fall within connectors and module hubs play an important role between and within modules (Ya et al., 2021). DI recorded the highest number of network edges with more complex structures and the interaction between AOA communities was closer. The high connectivity within the AOA community in DI results in higher complexity and stability, thereby maintaining the versatility and sustainability of the ecosystem. In addition, the interaction between AOB communities was closer under MF due to the high number of network edges which made the structure more complex. This indicated that MF had better connectivity and higher energy transfer efficiency between AOB communities.

## 5 Conclusion

This study focused on characterizing soil physicochemical properties, soil ammoxidation-related enzyme activities, AOA and AOB abundance, and community structure under three different irrigation methods, and also explored the response of AOA and AOB communities to the three irrigation methods using symbiotic networks. The results showed that DI and MF significantly increased the contents of AN, NO_3_^–^-N, moisture, and the activities of AMO and HAO in soil. Compared with FP, both water-saving irrigation methods (DI and MF) could increase the abundance of AOM, and DI had a more significant effect on the abundance of soil AOA and AOB. Different irrigation methods also significantly affected the community structure of AOM. Moreover, AN and moisture were the main driving factors affecting the changes in AOA and AOB communities, respectively, and the sensitivity of the AOB community to irrigation methods was higher than that of the AOA community. The network complexity of AOB was higher than that of AOA, indicating that the bacteria in the AOB community had a closer synergistic relationship and better-improved resource utilization efficiency and biological metabolic rate. The AOB community under MF had higher complexity and stability, and MF had a positive impact on the community structure of AOB.

## Data availability statement

The datasets presented in this study can be found in online repositories. The names of the repository/repositories and accession number(s) can be found below: https://www.ncbi.nlm.nih.gov/, SRP479978.

## Author contributions

RQ: Data curation, Writing – original draft, Methodology. MW: Funding acquisition, Writing – review and editing, Project administration. QL: Software, Writing – original draft. YL: Data curation, Writing – original draft. CL: Formal analysis, Writing – review and editing. JZ: Validation, Writing – review and editing. HL: Conceptualization, Writing – review and editing.
