# Supplementary material for: The different responses of AOA and AOB communities to irrigation systems in the semi-arid region of Northeast China
Source: Front Microbiol. 2024 May 7;15:1374618. doi: 10.3389/fmicb.2024.1374618 (PMC11106429; doi:10.3389/fmicb.2024.1374618)
Supplement: Supplementary file 1 [file Data_Sheet_1.docx]

| **TABLE S1.** Illumina MiSeq sequencing datas (at 97% sequence similarity) based on the AOA *amoA gene* | | |
| --- | --- | --- |
| Treatment | quality sequence | Final tag otu |
| FP1 | 80894 | 153 |
| FP2 | 90501 | 134 |
| FP3 | 78200 | 132 |
| DI1 | 103091 | 157 |
| DI2 | 77836 | 127 |
| DI3 | 100421 | 140 |
| MF1 | 115274 | 144 |
| MF2 | 75159 | 137 |
| MF3 | 77838 | 132 |

FP, traditional flooding irrigation; DI, shallow drip irrigation;

MF, mulching drip irrigation.

| **Table S2.** Illumina MiSeq sequencing data (at 97% sequence similarity) based on the AOB *amoA* gene | | |
| --- | --- | --- |
| Treatment | quality sequence | Final tag otu |
| FP1 | 40974 | 241 |
| FP2 | 56157 | 265 |
| FP3 | 48421 | 253 |
| DI1 | 47961 | 277 |
| DI2 | 43846 | 295 |
| DI3 | 41371 | 265 |
| MF1 | 33440 | 249 |
| MF2 | 37216 | 272 |
| MF3 | 35941 | 311 |

FP, traditional flooding irrigation; DI, shallow drip irrigation;

MF, mulching drip irrigation.

| **Table S3.** The BLAST of AOA at NCBI | | | | | |
| --- | --- | --- | --- | --- | --- |
| OTU | Taxonomy | Identities (%) | Bit score | E-value | Accession NO. |
| OTU_40 | [uncultured crenarchaeote](https://blast.ncbi.nlm.nih.gov/Blast.cgi#alnHdr_194465032) | 100 | 671 | 0 | [EU671980.1](https://www.ncbi.nlm.nih.gov/nucleotide/EU671980.1?report=genbank&log$=nucltop&blast_rank=11&RID=M3EU53D201N) |
| OTU_43 | uncultured Nitrososphaerota | 100 | 675 | 0 | KM595445.1 |
| OTU_42 | uncultured Nitrososphaerota | 99.7 | 671 | 0 | KM595440.1 |
| OTU_39 | uncultured Nitrososphaerota | 97.8 | 632 | 2.00E-176 | KM595556.1 |
| OTU_44 | uncultured Nitrososphaerota | 97.8 | 619 | 1.00E-172 | KY309238.1 |
| OTU_90 | uncultured Nitrososphaerota | 99.2 | 654 | 0 | KY130161.1 |
| OTU_45 | uncultured Nitrososphaerota | 94.8 | 573 | 2.00E-158 | KC735538.1 |
| OTU_138 | uncultured Nitrososphaerota | 97.8 | 619 | 1.00E-172 | KY308466.1 |
| OTU_46 | uncultured crenarchaeote | 100 | 675 | 0 | KY118350.1 |
| OTU_186 | uncultured crenarchaeote | 97.3 | 616 | 2.00E-171 | EU672223.1 |
| OTU_49 | uncultured crenarchaeote | 100 | 675 | 0 | KY118430.1 |
| OTU_47 | uncultured crenarchaeote | 98.9 | 652 | 0 | KC137432.1 |
| OTU_80 | uncultured crenarchaeote | 99.5 | 662 | 0 | EU672067.1 |
| OTU_48 | uncultured crenarchaeote | 100 | 675 | 0 | KY118429.1 |
| OTU_137 | uncultured crenarchaeote | 98.62 | 643 | 7.00E-180 | EU671586.1 |
| OTU_50 | uncultured crenarchaeote | 99.2 | 656 | 0 | KM000766.1 |
| OTU_52 | uncultured crenarchaeote | 100 | 675 | 0 | JQ260679.1 |
| OTU_120 | uncultured crenarchaeote | 97.5 | 616 | 2.00E-171 | EU672223.1 |
| OTU_62 | uncultured crenarchaeote | 99.5 | 664 | 0 | EU671823.1 |
| OTU_55 | uncultured crenarchaeote | 100 | 675 | 0 | EU672187.1 |
| OTU_160 | uncultured crenarchaeote | 98.6 | 643 | 7.00E-180 | KY807807.1 |
| OTU_53 | uncultured crenarchaeote | 99.2 | 658 | 0 | KY807798.1 |
| OTU_54 | candidatus Nitrosocosmicus | 99 | 475 | 3.00E-129 | CP017922.1 |
| OTU_136 | uncultured crenarchaeote | 98.35 | 640 | 9.00E-179 | EU672132.1 |
| OTU_64 | uncultured crenarchaeote | 100 | 673 | 0 | KY118422.1 |
| OTU_59 | uncultured Nitrososphaerota | 99.5 | 664 | 0 | KM087295.1 |
| OTU_76 | uncultured crenarchaeote | 100 | 673 | 0 | HM346069.1 |
| OTU_143 | uncultured crenarchaeote | 97 | 612 | 2.00E-170 | EU672223.1 |
| OTU_56 | uncultured Nitrososphaerota | 100 | 675 | 0 | JQ638729.1 |

| **Table S4.** The BLAST of AOB at NCBI | | | | | |
| --- | --- | --- | --- | --- | --- |
| OTU | Taxonomy | Identities (%) | Bit score | E-value | Accession NO. |
| OTU_100 | uncultured Nitrosomonadales | 100 | 813 | 0 | MH589282.1 |
| OTU_95 | uncultured *Nitrosospira* sp. | 99 | 793 | 0 | KP212533.1 |
| OTU_102 | uncultured *Nitrosospira* sp. | 99 | 816 | 0 | KP212550.1 |
| OTU_53 | uncultured Nitrosomonadales | 98 | 797 | 0 | MT416061.1 |
| OTU_103 | uncultured *Nitrosospira* sp. | 100 | 818 | 0 | KP212479.1 |
| OTU_114 | *Nitrosospira* | 97.8 | 780 | 0 | AY123836.1 |
| OTU_146 | uncultured *Nitrosospira* | 99.8 | 830 | 0 | KP212490.1 |
| OTU_121 | uncultured *Nitrosospira* sp. | 98.7 | 789 | 0 | KP212673.1 |
| OTU_111 | *Nitrosomonas* | 93.6 | 676 | 0 | AY123818.1 |
| OTU_183 | *Nitrosospira* | 96.7 | 752 | 0 | DQ228457.1 |
| OTU_52 | *Nitrosovibrio* | 97 | 791 | 0 | DQ228466.1 |
| OTU_108 | uncultured *Nitrosospira* sp. | 100 | 818 | 0 | KP212608.1 |
| OTU_107 | uncultured *Nitrosospira* sp. | 99.6 | 809 | 0 | KP212465.1 |
| OTU_113 | uncultured Nitrosomonadales | 98.7 | 804 | 0 | MH589275.1 |
| OTU_120 | uncultured Nitrosomonadales | 95.8 | 730 | 0 | MT416059.1 |
| OTU_115 | uncultured *Nitrosospira* | 99.3 | 821 | 0 | MW461459.1 |
| OTU_409 | *Bradyrhizobium* | 94.6 | 200 | 2.00E-46 | CP064693.1 |
| OTU_105 | *Nitrosomonas* | 92 | 636 | 2.00E-177 | AY123818.1 |
| OTU_149 | uncultured Nitrosomonadales | 96.7 | 752 | 0 | MT416044.1 |
| OTU_116 | uncultured *Nitrosospira* | 100 | 837 | 0 | KP212468.1 |
| OTU_119 | uncultured Nitrosomonadales | 100 | 837 | 0 | MH589285.1 |
| OTU_187 | uncultured Nitrosomonadales | 98.2 | 791 | 0 | MK822108.1 |
| OTU_122 | *Nitrosospira* sp. | 92 | 647 | 1.00E-180 | AY123832.1 |
| OTU_129 | uncultured *Nitrosospira* | 97.8 | 780 | 0 | MW461424.1 |

| **Table S5.** The explanation and contribution of RDA of soil physicochemical properties and AOA community under different irrigation methods (%) | | | |
| --- | --- | --- | --- |
| Factors | Explanation | Contribution | *P* |
| AN | 44.5 | 47.3 | 0.044 |
| Moisture | 29.5 | 31.4 | 0.024 |
| pH | 5.5 | 5.9 | 0.272 |
| SOM | 6.2 | 6.6 | 0.222 |
| NH_4_^+^-N | 5.3 | 5.6 | 0.244 |
| NO_3_^-^-N | 2 | 2.2 | 0.59 |
| TN | 0.9 | 1 | 0.868 |

SOM, soil organic matter; TN, total nitrogen; AN,

alkaline hydrolyzed nitrogen; NH_4_^+^-N, ammonium nitrogen;

NO_3_^-^_-_N, nitrate nitrogen.

| **Table S6.** The explanation and contribution of RDA of soil physicochemical properties and AOB community under different irrigation methods (%) | | | |
| --- | --- | --- | --- |
| Factors | Explanation | Contribution | *P* |
| Moisture | 24.6 | 26.2 | 0.032 |
| NH_4_^+^-N | 19.5 | 20.8 | 0.044 |
| TN | 13.7 | 14.6 | 0.214 |
| SOM | 7.2 | 7.7 | 0.504 |
| AN | 11.6 | 12.4 | 0.294 |
| pH | 10.2 | 10.9 | 0.312 |
| NO_3_^-^-N | 7 | 7.4 | 0.468 |

SOM, soil organic matter; TN, total nitrogen; AN,

alkaline hydrolyzed nitrogen; NH_4_^+^-N, ammonium nitrogen;

NO_3_^-^_-_N, nitrate nitrogen.


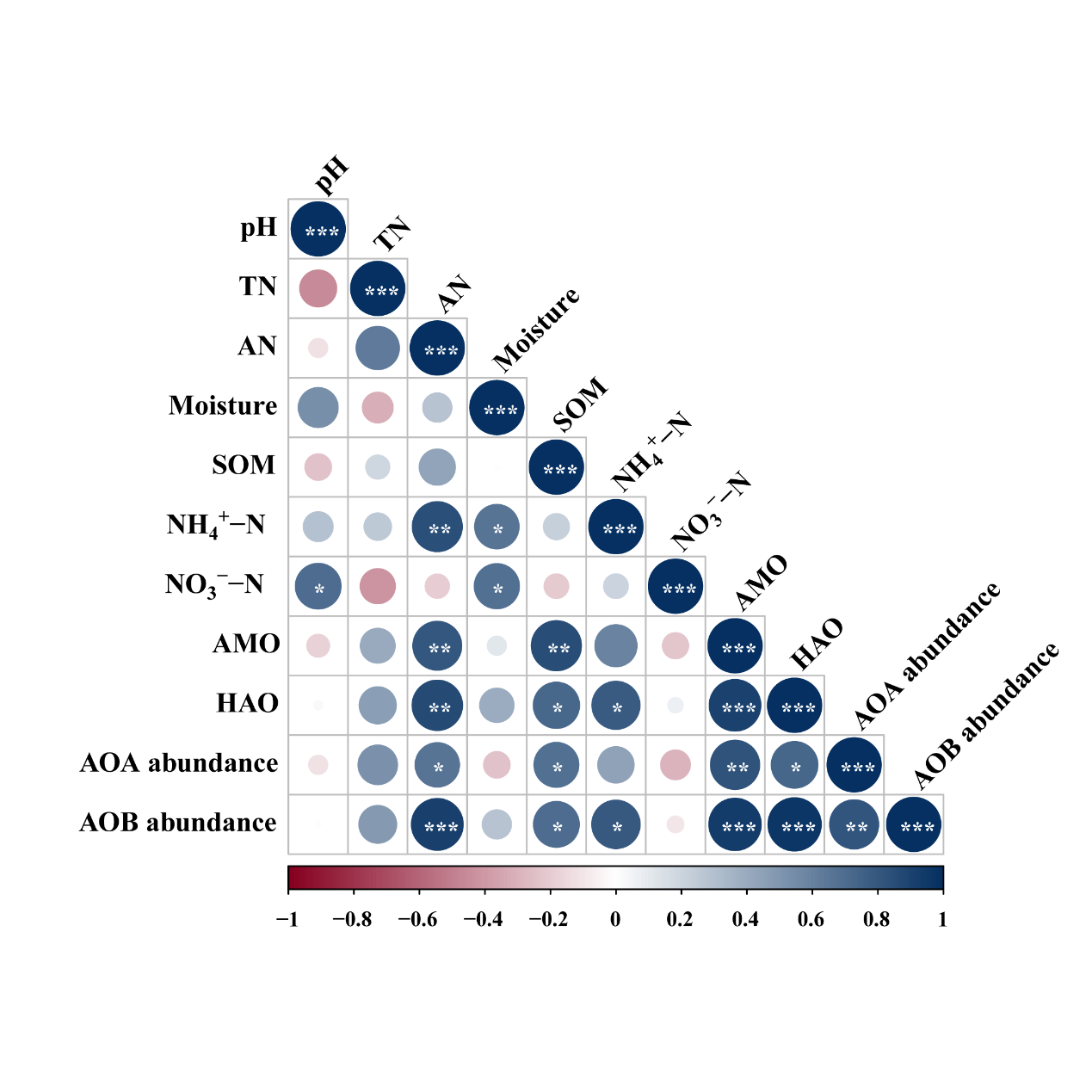


**Figure S1.** The correlation among soil properties, AMO, HAO activities and abundance of AOA *amoA*, AOB *amoA* genes. *, **, and *** represent the significant correlations at *P*<0.05, *P*<0.01, and *P*<0.001. SOM, soil organic matter; TN, total nitrogen; AN, alkaline hydrolyzed nitrogen; NH_4_^+^-N, ammonium nitrogen; NO_3_^-^-N, nitrate nitrogen. AMO, ammonia monooxygenase; HAO, hydroxylamine oxidase.

**Figure S2.** Venn diagram of AOM communities (A) is AOA, B) is AOB) under different irrigation methods. FP, traditional flooding irrigation; DI, shallow drip irrigation; MF, mulching drip irrigation.
